# Prescriptions

**Published:** 1896-09

**Authors:** 


TYPHOID DIARRHŒA AND TYMPANITIS.

℞. Acidi Carbolici.........gr. 1-6.
Ext. Opii.............. .gr. 1-6.
Bismuth Subnitrat......gr. iij
Ft. pilula. Sig.: [One pill of this formula three times a day.—*Dr. A. Hudson.*

OBSTINATE CASES OF CHRONIC MALARIA.

℞. Fl. ext. Eucalyptus Globulus....................
Glycerine..... ..........
Symple Syrup...........aa ℥iv.
M. Sig.: One teaspoonful once in four hours.—*Dr. D. E. Smith.*

A PROPHYLACTIC FOR CHILDREN AGAINST DIPHTHERIA.

℞. Potas. Chlor..........
Sod. Sulphitis.........aa gr. xv.
Acidi Muriatici........gtts. viij
Aquæ puræ..... .....℥ij.
Syr. simpl..... . .....℥ij.
M. Sig.: A teaspoonful twice daily.—*C. T. Poe, M. D.*

INDURATED GLANDS FOLLOWING SCARLET FEVER.

℞. Calcii Sulphid ..........gr. j.
Sacchar. Lactis.............gr. x.
M Ft. pulv. x et sig. One powder every hour or two.—*Dr. S. Ringer.*

NERVOUS DYSPEPSIA.

℞. Sodii Bromidi.............℥j.

Pepsin Sacch.............

Lig. Carb. Pulv... .....aa ʒiij.

Aquæ...... ..............ʒiv

M. Sig.: A teaspoonful three times a day in water after meals.—*Dr. Wm. A. Hammond.*

GOUT.

℞. Vini Sem. Colchici.......℥ss.

Potass. Iodidi............ʒij.

Liq. Potass...............℥ij.

Tinct. Zingiberis.........℥iss.

M. Sig.: A teaspoonful twice daily in warm water.—*Mr. Hodgson.*

CHRONIC RHEUMATISM.

℞. Pulv. Guaiaci... ........ʒij.

Pulv. Rhei.................ʒj.

Sulph. Subl.......... ....ʒij.

Potass. Nitrat ............ʒij.

Syr. Papaveris, q. s.

Ft. electuarium. Dose, ʒss ad ʒj.—*Middlesex Hospital.*

A VALUABLE ALTERATIVE PILL.

℞. Ext. Berberis Aquifolium, gr. ij.
Ext. Cascara Sagrada.... gr j.
M. Ft. pilula.

